# Electrochemical Synthesis of Mesoporous Alumina as an Adsorbent of Corrosion Inhibitors for Active Corrosion Protection in Organic Coatings

**DOI:** 10.3390/ma18184375

**Published:** 2025-09-19

**Authors:** Abenchara M. Betancor-Abreu, Javier Izquierdo, Raquel Rodríguez-Raposo, Ricardo A. Liria-Romero, Juan J. Santana, Ricardo M. Souto

**Affiliations:** 1 Department of Chemistry, Universidad de La Laguna, P.O. Box 456, 38200 La Laguna, Spain; abetanco@ull.edu.es (A.M.B.-A.); jizquier@ull.edu.es (J.I.); rrraposo@ull.edu.es (R.R.-R.); 2 Institute of Material Science and Nanotechnology, Universidad de La Laguna, 38200 La Laguna, Spain; 3 Department of Process Engineering, University of Las Palmas de Gran Canaria, 35017 Las Palmas de Gran Canaria, Spain; ricardo.liria@ulpgc.es

**Keywords:** active corrosion protection, mesoporous alumina, organic coatings, mild steel, electrochemical synthesis

## Abstract

This work describes a simple and economical electrochemical route for the generation of mesoporous alumina (MA) particles that can serve as containers for corrosion inhibitors for the active corrosion protection elements of metals when dispersed in organic coatings. The synthesis of precursor slurries was carried out in an electrochemical reactor with aluminum electrodes operating alternately as anodes and cathodes to facilitate metal dissolution and prevent passivation of the electrode surface. The obtained slurries were thermally treated to produce mesoporous alumina particles with adsorbent characteristics suitable for loading corrosion inhibitors. Benzotriazole (BTA) and 8-hydroxyquinoline (8HQ) were chosen as corrosion inhibitors. Dispersed in a commercial polymer matrix and applied to the coating of mild steel samples, the loaded MA improved the corrosion resistance of the coated metal exposed to a simulated marine environment. When physical damage is intentionally caused to expose the underlying metal, the polymer matrix containing BTA-loaded alumina particles retards the corrosion process due to the swelling of the inhibitor from the particles to the exposed bare metal in the scratch. Electrochemical impedance spectroscopy (EIS) measurements showed a marked increase in low-frequency impedance in coatings containing alumina particles, with the BTA-loaded system providing the most durable protection over extended immersion times (with a 50% improvement in corrosion resistance of steel exposed within the scratch). This demonstrates the potential of this approach for long-term corrosion protection applications.

## 1. Introduction

Metals conduct electricity and heat relatively well. Once transformed, raw materials produce metallic materials that are widely used in industry and technology. However, despite their use, metals in their pure state are thermodynamically unstable and easily degrade through a phenomenon called corrosion [1]. During corrosion processes, the dissolution of the metal and the degradation of its properties occur due to the physicochemical interaction of the metal with the environment [2,3]. This is a favored process [4] and, therefore, an inevitable phenomenon [5]. While this phenomenon is of great importance for metallic materials, all materials can corrode. Therefore, the phenomenon of corrosion remains the main obstacle for industries worldwide [6]. This renders corrosion science a topic of utmost importance to the scientific community, directed to understand and prevent the huge economic impact that this phenomenon has on our society (about 3.5% of the world’s Gross Domestic Product (GDP)) [1]. Besides the economic factor, these chemical processes bring other repercussions: safety, environmental and technological problems, as well as efforts dedicated to the study and control of the corrosive process [1,5]. Corrosion is also an electrochemical phenomenon (among others) and, therefore, electrochemical methods are the most effective tools for its study [4,6].

The use of coatings is the most widespread method to protect metal parts from corrosion [7,8]. Typically, these coatings consist of a polymer matrix adhered to the metal surface, acting as a barrier against electrolyte diffusion and permeability [7]. Their effectiveness depends on adhesion to the surface, thickness, dielectric properties, additives, condition of the metal surface, ambient exposure conditions, reactions occurring at the interface of the coating and the metal, etc. Although new coating systems are constantly being developed, a technological interest lies in improving the coatings available on the market to provide a more robust and effective system against corrosive processes. One strategy for this is the incorporation of micro- and nanoscale additives into the matrix to produce smart coatings [9]. During curing, solvent volatilization inevitably generates micropores and defects that can allow corrosive species to reach the substrate and initiate corrosion. The addition of these particles improves the integrity of the coating by filling the micropores and hindering the diffusion of corrosive species, thus delaying their access to the substrate, and thereby reducing degradation phenomena such as blistering, delamination or cracking [10]. According to the IUPAC definition, a mesopore is an intermediate-sized pore. Pores wider than approximately 0.05 μm (50 nm) are classified as macropores, while those not exceeding approximately 2 nm are referred to as micropores [11]. Consequently, the mesoporous range is defined as pores larger than 2 nm but smaller than 50 nm in diameter. This structural regime provides a balance between high surface area and accessible pore channels, which is particularly advantageous for these types of applications.

Among the various inorganic oxide nanoparticles used for protective coatings, mesoporous alumina (MA) offers a unique combination of properties that make it particularly attractive. Like other oxides (SiO_2_, TiO_2_, ZnO, Fe_2_O_3_), alumina contributes to improving the barrier properties of epoxy matrices, but it is distinguished by its intrinsic hardness, high thermal conductivity, mechanical strength, abrasion and scratch resistance [12], and its remarkable chemical stability in both acidic and alkaline environments [12,13]. Its low processing cost (such as electrocoagulation process), non-toxicity and easy availability further strengthen its suitability as a large-scale additive [11,12].

Compared with titania, which is highly valued for its photocatalytic behavior under UV illumination, alumina offers a more stable anticorrosive effect in the dark or indoor conditions, where TiO_2_ loses much of its protective capacity [14]. Similarly, although mesoporous silica is widely studied due to its large surface area and well-defined porous structure, it often exhibits instability in aggressive environments such as concentrated saline or acidic media [15]. On the other hand, zinc oxide nanoparticles can act as sacrificial anodes, but their partial solubility in chlorinated media compromises their long-term durability, unlike alumina, which remains chemically stable under such conditions [16].

In addition, these particles can retain corrosion inhibitors that significantly increase their anti-corrosion effectiveness. These smart coatings can produce the selective release of inhibitory substances in response to chemical stimuli causing the degradation of the metal substrate, thus reducing the risk of leaching of these chemicals, which would limit the environmental problems associated with their use [17].

In this context, smart coatings offer an interesting application for functionalized mesoporous alumina nanoparticles. These materials are designed to adaptively respond to the degradation of metal substrates by selectively releasing corrosion inhibitors when needed. One of their main advantages is the controlled release of inhibitors, which reduces the environmental impact compared to traditional coatings. Organic compounds such as benzotriazole (BTA) and 8-hydroxyquinoline (8HQ) have demonstrated remarkably high efficacy as corrosion inhibitors by forming protective films on metal surfaces [18]. BTA, a heterocyclic compound with solubility in aqueous media, can disperse and adsorb efficiently onto metal substrates. Its ability to form a stable and adherent film provides a robust barrier against corrosive agents, making it particularly effective in aggressive environments [19,20]. On the other hand, 8HQ is well known for its coordination chemistry, owing to the presence of nitrogen and oxygen atoms, as well as benzoyl rings, which enhance its adsorption capacity and inhibitory effect [21]. Due to these properties, 8HQ and its derivatives are widely used as corrosion inhibitors for steels in various aqueous solutions. These characteristics promote strong interactions with metal surfaces, allowing 8HQ to act as a mixed-type inhibitor that forms stable protective layers and improves corrosion resistance [22,23]. This confirms that anodic dissolution of steel and cathodic hydrogen evolution are reduced in the presence of the 8HQ inhibitor [24]. In addition to the inhibitory effect of these compounds, studies have also reported improved adhesion when inhibitor-loaded particles are incorporated into coatings, for example, in systems containing 8HQ [24]. In the particular case of BTA, its ability to form a stable adsorbed layer at the metal-coating interface has been reported to enhance interfacial bonding. In this case, the azole groups of BTA promote the formation of an iron–azole interfacial layer that acts as an adhesion promoter, strengthening the interaction between the polymer coating and the steel surface [25]. For this reason, BTA is a well-known inhibitor for steel [26], whose effectiveness has been consistently demonstrated for over 30 years [27].

However, it is important to note that the improved adhesion observed in coatings containing inhibitor-loaded MA cannot be attributed solely to the presence of mesoporous alumina. A more precise interpretation is to consider this feature as a synergistic effect that involves the preparation of the substrate, which guarantees an adequate adhesion base; the mesoporous alumina, whose high hardness and large specific surface area improve mechanical and barrier properties; and the specific chemical composition of the inhibitors, which provides additional interfacial interactions; between other factors.

The synthesis of mesoporous alumina particles can be carried out by various established methods, such as sol–gel technique, precipitation methods, spray pyrolysis, laser ablation, microwave-assisted solvothermal processes, and combustion synthesis [28]. These techniques are well-documented and widely applied in materials science. However, most methods involve multiple synthesis steps, specialized equipment, and energy-intensive protocols. In the face of growing environmental concerns, current research increasingly favors sustainable synthesis routes. These aim to minimize the use of toxic precursors, reduce or eliminate organic solvents, simplify reaction steps, and limit waste generation [20]. Among these approaches, the sol–gel method [18] stands out for its low cost and simplicity of implementation. It allows the formation of homogeneous alumina products at relatively low temperatures. However, this method presents challenges such as particle agglomeration, expensive storage conditions, and the need for careful handling procedures [29]. Alternative methods also have disadvantages. For example, the combustion method is a rapid process that produces high-purity materials but requires elevated temperatures. The hydrothermal technique, although it can produce less aggregated particles, requires expensive and complex equipment [19,28]. In contrast, mechanical synthesis by ball milling has a simpler setup but may introduce unwanted impurities due to prolonged mechanical stress [19]. Given these limitations, the design of materials with controlled porosity is essential to optimize surface properties for applications in catalysis, adsorption and corrosion protection [22,29].

Regardless of the method chosen for the synthesis of mesoporous alumina, it generally aims to improve the surface properties through larger specific surface areas than conventional alumina, a narrower pore size distribution, and the absence of micropores. To overcome the limitations of conventional synthesis methods, recent research is exploring alternative routes such as electrochemical synthesis. Techniques such as electrocoagulation (EC) have shown promise due to their relative simplicity and low environmental impact [30]. However, the literature on the use of electrochemical methods for alumina production [23] is still limited, and little is known about how operating parameters such as current density, electrocoagulation time, pH, and post-treatment steps affect the morphological and structural characteristics of the resulting alumina [30].

This study proposes a simple and economical procedure for the synthesis of alumina as a functional support for BTA and 8HQ in the development of active anticorrosion coatings using an electrochemical route. The obtained alumina was functionalized with both inhibitors and integrated into a polymer matrix. The resulting particles were characterized using scanning electron microscopy (SEM) coupled with energy dispersive X-ray spectroscopy (EDS) for surface analysis, while the corrosion behavior of the modified coating systems was analyzed by various electrochemical techniques, namely electrochemical impedance spectroscopy (EIS) [31], scanning vibrating electrode technique (SVET) [32], and scanning electrochemical microscopy (SECM) [33].

## 2. Materials and Methods

**Figure 1:**
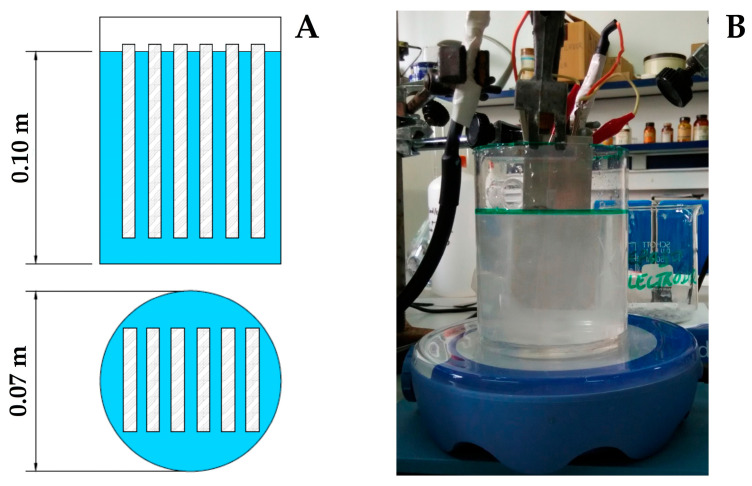
Bipolar electrochemical cell employed for the alumina synthesis: (**A**) Sketch describing the parts and electrical connections; and (**B**) photograph taken during the actual operation of the cell.

Commercial aluminum plates of 50 mm × 120 mm × 2 mm were used as electrodes in the electrochemical reactor for alumina synthesis. Before use, they underwent a cleaning and conditioning process according to ASTM G1-03(2017) [34], including light mechanical cleaning (to remove surface contaminants), followed by chemical cleaning in a mild NaOH solution to displace strongly adsorbed surface material. The electrodes were then thoroughly rinsed with deionized water and finished with a solvent rinse to remove residual organics, followed by drying. This sequence ensured effective removal of corrosion products and contaminants without damaging the metallic substrate. The electrochemical reactor was adapted from [35]. It was built around a cylindrical vessel with an effective volume of 368 mL, containing six aluminum plates with a total active area of 147 cm^2^, placed parallel to each other 5 mm apart, and filled with tap water. In this way, a surface/volume ratio close to 0.4 was achieved. Magnetic stirring was continuously applied at the bottom of the reactor. Figure 1 shows the resulting assembly. The electrochemical cell was powered by an AIM-TTI QPX1200SP power supply (TTI Europe, Maisach-Gernlinden, Germany), allowing the current supplied to the system to be controlled. The overall process and system control were managed by a Windows-based PIPO computer (PiPO Technology, Shenzhen, China) and a custom control software interface to regulate the current density at 5 mA/cm^2^. The polarity of the electrodes was reversed every 5 min, so that both electrodes functioned as cathode or anode for the same period of time and, therefore, consumed to an almost equal extent during the experiments.

The pH was measured at the beginning of the experiments (pH = 7.11) and at 10 min intervals, reaching a value of 7.85 after one hour. With the current density being fixed, the potential varied with time and half-period, showing an increase of 0.41 V during one hour of operation. The specific energy consumption (*SEC*) of the electrocoagulation process was calculated according to:

(1) $$SEC=\frac{U\cdot I\cdot t}{V}$$

where *U* is the applied voltage (V) provided by the DC power supply, *I* is the applied current (A), *t* is the electrocoagulation time (h), and *V* is the treated solution volume (m^3^). The voltage was monitored from the power supply output, the current was set at the desired fixed value, and the treatment time was recorded during each experiment. The measured energy consumption during the process was 0.80 kWh/m^3^, which is in line with literature values such as 0.57 kWh/m^3^ [36], 0.72 kWh/m^3^ [37], or up to 4 kWh/m^3^ [38]. However, this value can vary depending on variables such as the electrolyte composition, electrode spacing, current density, and other operational parameters.

The sludge was collected by filtration and then oven-dried for 18 h at 80 °C. It was then heat-treated in a muffle furnace at 650 °C for 6 h under controlled atmosphere to remove volatile components present in the dried sludge. Finally, the material was ground and stored in an oven at 50 °C until further use.

The metallic substrates used were mild steel. Prior to coating, the surfaces underwent a pickling pretreatment. The samples were first sanded using a Bosch PSM100 W corded multi-sander (Bosch, Gerlingen, Germany), equipped with 120-grit sandpaper. They were then washed with soap and water to remove any grease residue. To remove detergent residue, the samples were then rinsed with MilliQ-grade water. Finally, to ensure complete surface drying and remove any residual moisture, they were rinsed with acetone.

The electrolyte for the corrosion test, a 0.5 M NaCl aqueous solution, was prepared using analytical-grade chemicals and Milli-Q^®^ deionized water (Millipore, Burlington, MA, USA). NaCl and the corrosion inhibitors 8-hydroxyquinoline and benzotriazole were supplied by Sigma Aldrich (Saint Louis, MO, USA).

A commercial organic coating used in the marine industry served as the polymer matrix. The chosen matrix was Hempel’s High Protect 35651 (Hempel, Kongens Lyngby, Denmark), a two-component, high-build, solvent-free epoxy coating. This coating is characterized by the formation of a hard, waterproof, and resistant film, offering good tolerance to environmental conditions. Its application requires a hardener (Curing Agent 97351), composed of hydroxyl-substituted molecular groups, such as benzyl alcohol and salicylic acid, among others. A mixing ratio of 3:2 by volume (resin/hardener) was used.

Electrochemical studies were carried out in a three-electrode cell equipped with a platinum counter electrode and a KCl-saturated silver/silver chloride (Ag/AgCl) electrode as a reference electrode. Measurements were conducted at the open-circuit potential (OCP) of the metal coating system in the test electrolyte. After stabilization of its OCP at approximately one hour, the electrochemical test was initiated. Electrochemical impedance spectroscopy (EIS) measurements were performed using a PARSTAT 2263 potentiostat/galvanostat (AMETEK Scientific Instruments, Berwyn, PA, USA) equipped with an internal frequency response analyzer. Impedance spectra were recorded at the OCP with a sinusoidal alternating voltage of amplitude ±20 mV in the frequency range from 10 kHz to 10 mHz, with 5 points per decade. SVET measurements were performed using an instrument from Applicable Electronics (New Haven, CT, USA). The Pt:Ir sensing probe, with a diameter of 10 µm, was coated with a spherical platinum black deposit. The tip scanned the surface in the *XY* plane at a distance of 100 μm whereas it was vibrated 20 μm in the Z direction with frequency 70 Hz; its operation was monitored using a video zoom camera placed vertically over the sample. SECM measurements were made with an instrument built by Sensolytics (Bochum, Germany), using a 25 µm diameter Pt disk microelectrode embedded in a glass capillary as the tip. The tip was polarized at −0.70 V vs. Ag/AgCl to monitor the consumption of dissolved molecular oxygen due to the cathodic process [39], while scanning at an approximate distance of 25 µm from the sample.

Surface characterization was performed by scanning electron microscopy (SEM) coupled with an Energy dispersive X-Ray spectrometer (EDS) using FESEM Zeiss Sigma 300VP equipment from Zeiss (Oberkochen, Germany). Surface area and pore size distribution were characterized using a Gemini V surface analyzer from Micromeritics (Norcross, GA, USA), employing the single-point method, also called the single-point BET.

## 3. Results and Discussion

First, the synthesis of alumina particles and their loading with corrosion inhibitors were characterized. The corrosion properties of mild steel coated with an anti-corrosion coating modified by alumina particles were then monitored by electrochemical methods.

### 3.1. Synthesis and Loading of Alumina Porous Particles

Anodizing aluminum electrodes in an initially neutral aqueous medium results in the dissolution of aluminum ions, which then undergo hydrolysis in the electrolyte. Since electrochemical oxidation generally results in the formation of a compact layer of alumina on the metal, with dielectric properties, thus preventing its complete dissolution by passivation, the main objective is to prevent the precipitation of alumina on the electrode surface. In other words, the hydroxyl complexes of the oxidized aluminum must be emulsified for subsequent coagulation. This is achieved by regularly changing the polarity of the electrodes so that they function as both cathodes and anodes. During this operation, the oxidation of the anode gives rise to metal ions:

(2) $$M\;\to M^{z+}+ze^{-}$$

where *z* = number of electrons transferred in this half-reaction.

On the other hand, the cathode generates hydroxyl ions and hydrogen gas

(3) $$H_{2}O+e^{-}\;\to \;\frac{1}{2}\;H_{2}+{OH}^{-}$$

**Figure 2:**
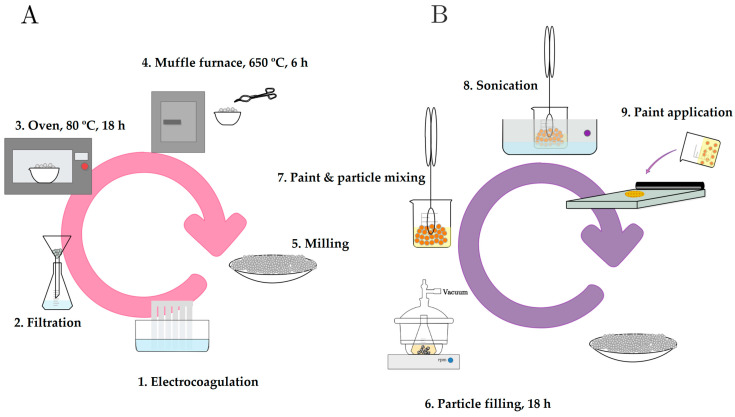
Schematic representation of the processes used for the fabrication and functionalization of mesoporous alumina particles. (**A**) Electrochemical synthesis of alumina by electrocoagulation, followed by filtration, drying, calcination, and milling to obtain mesoporous particles. (**B**) Loading of corrosion inhibitors (BTA and 8HQ) into the alumina structure by vacuum-assisted impregnation, allowing their use as active containers for anticorrosion coatings.

Electrolytic reactions take place on the electrode surface, resulting in the formation of metal hydroxides. When aluminum electrodes are used, various monomeric and/or polymeric metal hydroxides are formed: [Al(OH)]^2+^, [Al(OH)_2_]^+^, [Al_2_(OH)_2_]^+^, Al(OH)_3_, [Al_6_(OH)_15_]^3+^, [Al_7_(OH)_17_]^4+^, [Al_8_(OH)_20_]^4+^, [Al_13_O_4_(OH)_24_]^7+^, [Al_13_(OH)_34_]^5+^ [40]. Finally, the colloids can be separated by sedimentation/flotation techniques [41]. In our case, the sludges were separated by vacuum filtration treatment, and then dried in an oven for 18 h at 80 °C. The metal hydroxides can be converted into alumina (Al_2_O_3_) by calcination. This treatment is carried out in a muffle furnace at 650 °C for 6 h under a controlled atmosphere in order to eliminate the volatile components present in the dried sludge. Finally, the material underwent a milling process and was stored in an oven at 50 °C until further use. Figure 2A shows a sketch of the process employed for the synthesis of the alumina particles.

The surface area and porosity of the obtained MA particles were analyzed using the single-point method, also called single-point BET. The data in Table 1 show a pore size of 7.68 nm, corresponding to a mesoporous sample. The measured BET surface area was 231 m^2^/g, within the typical range of alumina samples (50–300 m^2^/g).

Regarding the particle loading process, it can be classified as an adsorption-based encapsulation method, where the active compounds are incorporated onto and into solid particles (specifically alumina) by impregnation of functional agents (8HQ and BTA) into the pores or surface of the material. Considering the available literature reports on the spectroscopic characterization of BTA loading on mesoporous silica nanoparticles [26,27,42,43] and 8HQ on mesoporous resorcinol-formaldehyde hollow nanospheres [44], it can be expected that the loading of corrosion inhibitors on MA particles is a predominantly physical adsorption process. In other words, it must be a surface-driven phenomenon, enhanced by the application of vacuum, which facilitates capillary-driven penetration and access of inhibitors to the porous structure. This would result in a heterogeneous multi-core structure, where the inhibitors are randomly distributed in the pores, interstitial spaces and the external surface of the alumina particles.

**Figure 3:**
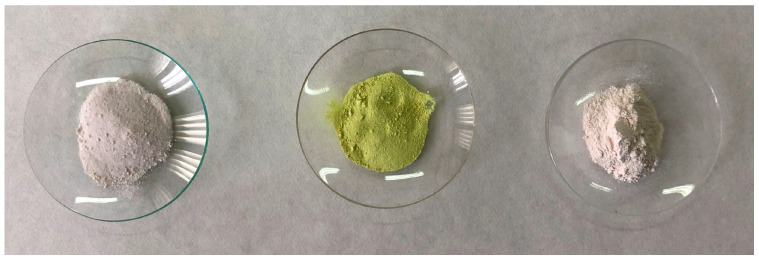
Photograph showing alumina preparations: unloaded (**left**), and loaded with 8-hydroxyquinoline (**center**) or benzotriazole (**right**).

Functionalization was performed by the following steps (see Figure 2B):

- Particle filling: Saturated solutions of 8-hydroxyquinoline and of benzotriazole in acetone were prepared, and they were separately added to pre-milled alumina samples. The system was subjected to stirring at a speed of 1000 rpm to ensure proper mixing of the solution with the alumina. This process was performed while applying vacuum to allow the saturated solution to effectively replace the air trapped within the capsules. Vacuum and continuous stirring were maintained for 18 h to promote thorough impregnation of the alumina with the solution.
- Filtration and Solid Processing: The solid was separated by filtration, followed by three washes with acetone. The obtained solid was further milled and stored in a furnace at 50 °C until use. Figure 3 shows a photograph of the alumina preparations, unloaded and loaded with 8HQ or BTA. A yellowish color of the inhibitor-loaded MAs is a direct consequence of the presence and surface distribution of the organic inhibitors on them. (In fact, the solution used in the filling step is already colored; the fact that the particles retain the same color as the solution is due to the loading by the inhibitor).

**Figure 4:**
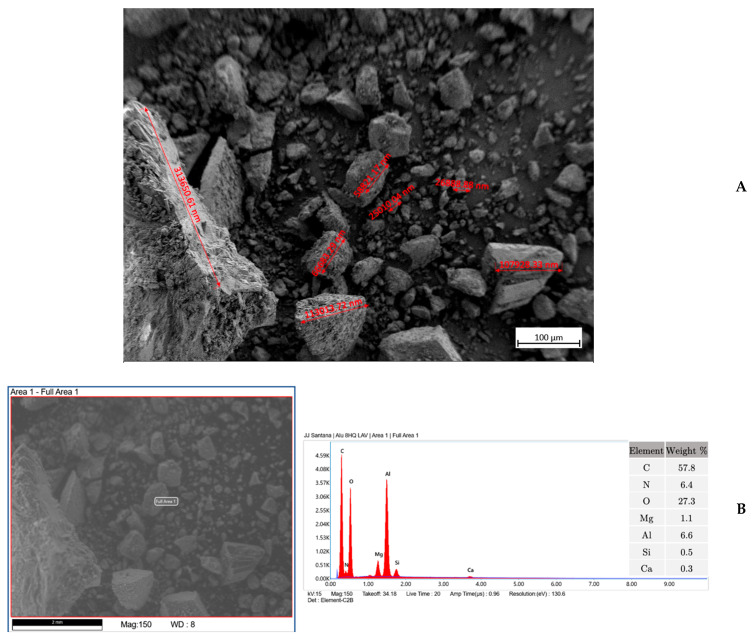
Characterization of the alumina sample loaded with 8HQ. (**A**) SEM micrograph acquired operating at 15 kV, that shows the morphology and particle size distribution of the alumina sample. (**B**) Corresponding EDS spectrum confirming the presence of nitrogen, indicating successful loading of 8HQ.

The inhibitor-loaded samples were analyzed by scanning electron microscopy (SEM). The results for 8HQ-loaded alumina (Figure 4A) show a rather heterogeneous particle distribution, with a predominance of small particles, although larger particles are occasionally observed. The presence of nitrogen in the sample, confirming the presence of the inhibitor, is clearly established by the EDS analysis in Figure 4B.

**Figure 5:**
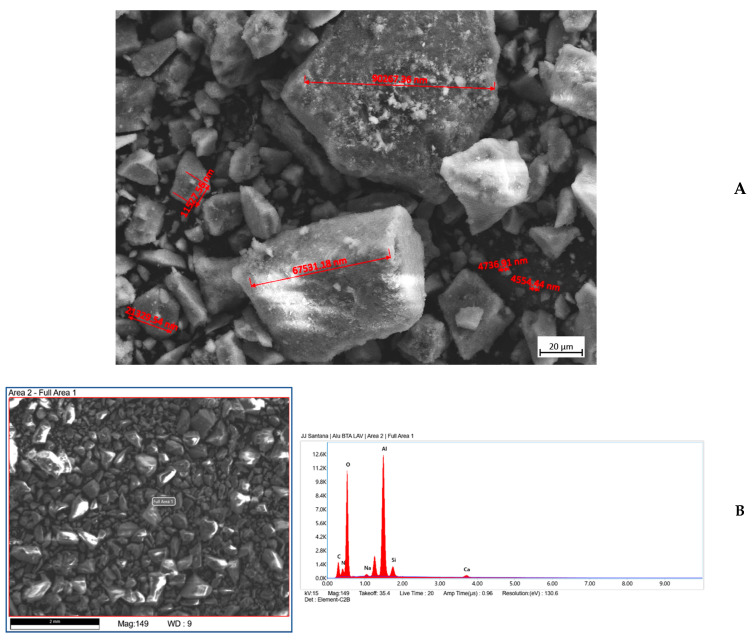
Characterization of the alumina sample loaded with BTA. (**A**) SEM micrograph acquired operating at 15 kV, that shows the morphology and particle size distribution of the alumina sample. (**B**) Corresponding EDS spectrum confirming the presence of nitrogen, indicating successful loading of BTA.

In the BTA-loaded alumina sample shown in Figure 5A, a particle size distribution similar to that of the 8HQ-loaded alumina is observed (cf. Figure 4A). However, although the particle size distribution appears very similar, in this case the largest particle sizes observed were approximately 100 µm. The EDS results shown in Figure 5B confirm the presence of the inhibitor.

A previous study by our group [45] showed that alumina particles can achieve inhibitor loadings above 35 wt.%, as determined by thermogravimetric analysis (TGA). In contrast, reports using other metal oxides as carriers exhibit lower capacities, such as 16 wt.% [42] and 21 wt.% for BTA-loaded SiO_2_ [43], and 33 wt.% for 8HQ [44]. Interestingly, higher loading values have been reported for BTA when using vacuum-impregnation techniques [46], highlighting that the final capacity is strongly influenced not only by particle type and morphology, but also by the encapsulation methodology.

**Figure 6:**
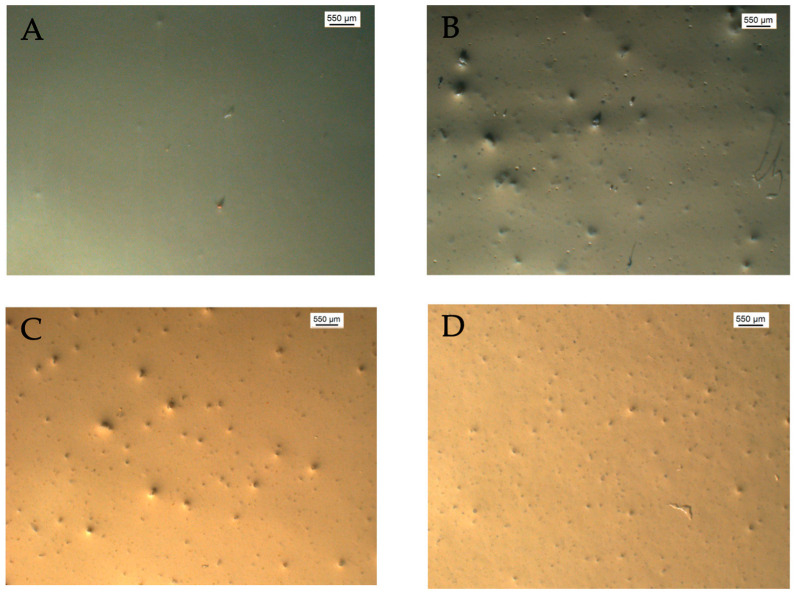
Optical microscope images showing the finished surface of the various painted mild steel samples considered in this work: (**A**) unmodified coating, (**B**) coating containing MA particles, and coatings containing MA particles loaded with 8HQ (**C**) or BTA (**D**).

Finally, mild steel samples were coated to a thickness of 160 µm with a two-component epoxy resin for corrosion protection. The alumina particles, with and without functionalization with a corrosion inhibitor, were added under continuous stirring to the mixture formed by the coating and the hardener before the application of the coating. Figure 6 shows optical micrographs of the resulting coated surfaces for the four systems considered: the unmodified epoxy coating, the epoxy coating filled with alumina particles without inhibitor load, the epoxy coating containing alumina particles loaded with 8HQ and that containing alumina particles loaded with BTA.

### 3.2. Electrochemical Characterization

The corrosion resistance of coated mild steel was studied in a 0.5 M NaCl aqueous solution, naturally aerated, at ambient temperature (nominally 20 °C). Prior to the corrosion test, a defect was cut through the coating using a scalpel blade to expose the underlying metal. The defect dimensions are shown in Table 2. This allowed us to observe the different capabilities of the coating systems to repair (at least partially) the corrosion process and better protect the mild steel surface exposed to the test electrolyte.

**Figure 7:**
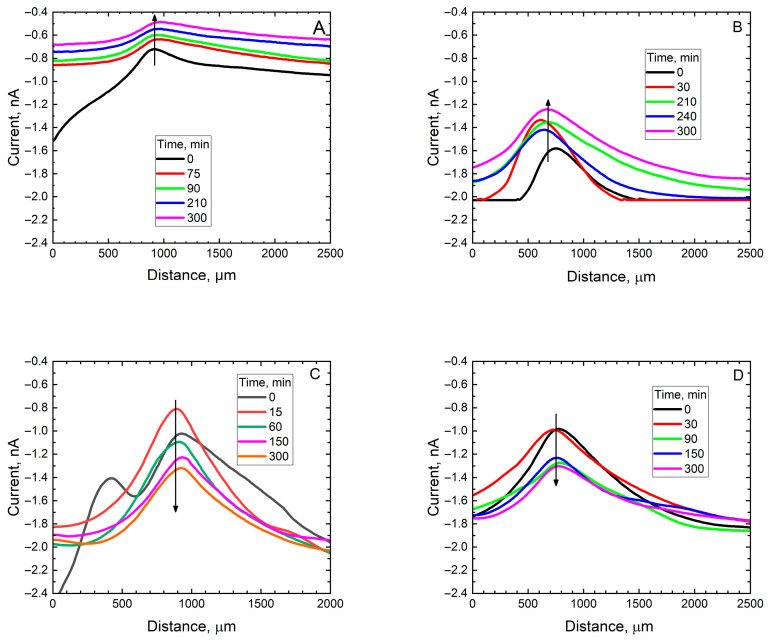
SECM scan lines taken across the scratch produced on mild steel substrates coated with a commercial epoxy coating: (**A**) unmodified, (**B**) containing alumina particles without corrosion inhibitor, (**C**) containing alumina particles loaded with 8HQ, (**D**) containing alumina particles loaded with BTA. The samples were exposed in 0.5 M NaCl for the different times indicated in the Figure. SECM tip: Pt, dia. 25 μm; *E*_tip_ = −0.70 V vs. Ag/AgCl; tip-substrate distance: 25 μm; scan rate 10 µm/s. The arrows are drawn to indicate the direction of change with time observed in the plots.

Moreover, the process is expected to operate on a very small scale until it can produce a surface modification noticeable in a larger scale. Therefore, corrosion initiation was initially studied by scanning electrochemical microscopy (SECM), a microelectrochemical technique combining chemical and spatial resolution. In this case, the tip of a platinum microelectrode is scanned in close proximity to the corroded sample to monitor any local variations in chemical or electrochemical reactivity due to the corrosion process. In this context, since the corrosion test is performed with the sample unpolarized, corrosion reactions begin when a local distribution of anodes and cathodes develops on the metal surface. While the anodic process leads to the dissolution of the metal, the electrons released by the oxidizing metal atoms must be consumed by a chemical species present in the environment at the cathodic sites. In our case, the oxidizing species is molecular oxygen dissolved in the aqueous electrolyte. Therefore, a local decrease in oxygen concentration will occur around the cathodic sites after the corrosion process begins. By biasing the tip of the platinum microelectrode at a potential of −0.70 V vs. Ag/AgCl, at which steady-state electroreduction of O_2_ ions to OH^−^ occurs, the presence of cathodic sites on the surface will be evidenced by sensing a smaller amount of oxygen available for electroreduction at the tip. In this work, the oxygen concentration distributions over the scratches produced to the four coating systems at different times were recorded and are graphically represented in Figure 7.

The progressive oxygen consumption resulting from the activation of the cathodic corrosion reaction in the scratch becomes clearly visible when observing the first scan line of Figure 7A, obtained immediately after the introduction of the electrolyte into the electrochemical cell for the steel coated with the unmodified coating. When the tip moves from left to right, a sharp decrease in the value of the oxygen electroreduction current measured at the tip is observed. Then, at the exit of the scratch, the decrease in the concentration of this molecular species due to diffusion towards the defect leads to lower currents than at the beginning of the scan line. Indeed, the currents measured at the end of the first scan, outside the scratch, are similar to those observed during the other scans, both at the beginning and at the end, on the coating. Furthermore, the currents measured in the gap remain low (about ¼ of the current measured at the beginning of the experiment), demonstrating that the corrosion process progresses steadily, with the exposed metal unable to passivate. The process of progressive depletion of dissolved oxygen remaining in the electrolyte in contact with the exposed steel in the defect occurs similarly in the case of the coating that includes alumina particles that have not been loaded with corrosion inhibitor, confirming that this system is neither capable of providing active protection to bare mild steel (see Figure 7B).

A different behavior is observed in the case of the scan lines measured for the coating functionalized with 8HQ, shown in Figure 7C. The first scan again shows the effect of a gradual and abrupt oxygen consumption in the scratch, as the linear scan progresses from left to right, following the activation of the corrosive process in the unprotected metal. Although the following scans present symmetrical curves corresponding to a decrease in the oxygen concentration in the scratch, it is worth noting that the curves gradually evolve towards higher current values (downward on the graph, corresponding to the direction of the increasing negative cathodic currents), indicating a gradual slowing down of the corrosive process, at least a few hours after its onset. Interestingly, such progressive inhibition of the oxygen consumption inside the scratch is accompanied by an approximately stationary quantity of oxygen observed outside the defect, since concentration gradients as large as those in Figure 7A,B do not occur. A similar effect also occurs in the case of the scan lines in Figure 7D which corresponds to the case of BTA loading, making it difficult to establish differences in inhibitor behavior based solely on SECM data.

**Figure 8:**
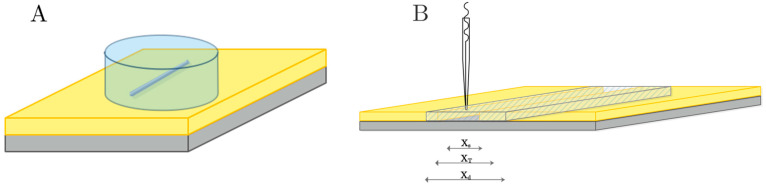
Schematics describing the measurement operation used in SECM: (**A**) A small electrochemical cell is constructed on the coated steel sample, leaving the scratched area in its center. (**B**) The measured area in the sample containing the scratch as the tip scans across the defect from left to right: X_s_ denotes the actual scratch width (50 μm); X_T_ includes the scratch width, its edges, and the corrosion product precipitation sites (200–500 μm) at the borders of the scratch; and X_d_ is the scanned distance covered by the tip to record one scan line (2000 μm).

To confirm that the current effect observed near the defect can be attributed to a decrease in oxygen concentration near the area scanned by the electrode, an alternative measurement was performed by SECM. As mentioned earlier, this phenomenon is considered to be due to the low diffusion rate of dissolved molecular oxygen from the solution to the scanned area near the scratch, which could result in a progressive decrease in the cathodic current values measured outside the defect over the coated steel [47]. The competition for oxygen and the relatively large size of the scratch compared to the scanned area could lead to a change in the limiting current measured at the microelectrode tip and, consequently, maintain a local oxygen depletion. This behavior is related to the fact that in SECM measurements, the current recorded at the microelectrode tip is limited by the diffusion rate of the redox mediator; moreover, the probe can also obstruct the diffusion of chemical species [48]. In summary, the scratched area can be considered significant compared to the scanned insulating surface, combined with a low diffusion rate, so that the local oxygen concentration decreases in the absence of an inhibitory effect. Figure 8A shows a schematic of the experimental setup used for SECM measurements, where the defect present in the sample can be observed. Figure 8B also shows the defect dimensions and the scanned area.

**Figure 9:**
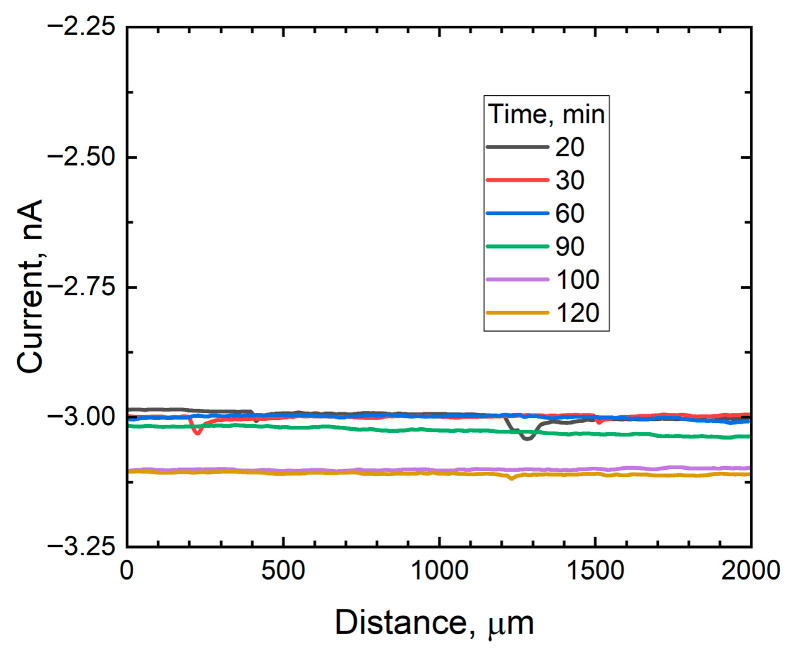
Scan lines recorded over a region of the intact coating by SECM. Scan rate: 10 μm/s, step width: 10 μm, applied potential: −0.70 V, and tip height: 50 μm.

To validate this hypothesis, successive scan lines were recorded on an inert surface (length = 2500 µm) and the behavior of the limiting current measured at the tip for oxygen electroreduction was recorded. Figure 9 shows a selection of the current graphs recorded over a period of 120 min. The results show that the potential applied to the microelectrode tip traveling at a short distance from the insulating surface does not significantly affect the local oxygen concentration, as the current signal remained practically stationary during the 120 min experiment.

**Figure 10:**
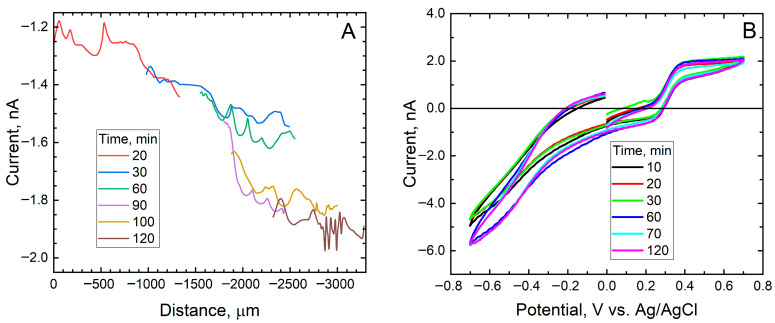
(**A**) Scan lines recorded on the real sample by SECM. Scan rate: 10 μm/s, step width: 10 μm, applied potential: −0.70 V, and tip height: 25 μm. (**B**) Voltammograms recorded using 0.5 mM FcMeOH added to the 0.5 M NaCl test solution. They were recorded after applying a potential of −0.70 V to the tip for recording the scan lines in (**A**).

In contrast, when measurements were taken over a scratch on the coated sample without functionalization (containing alumina particles without corrosion inhibitor), a variation in current was observed at the insulating surface. Figure 10A shows the tip response as it moves away from the defect along the X-axis. Scans were performed in different areas due to the irregularity of the sample surface, which made scanning over a large area difficult.

These measurements show higher current values at the defect and a gradual change in current as the tip moves away from it. This supports the hypothesis of a large O_2_ consumption by the sample and the inability of the solution to replenish oxygen near the defect due to slow oxygen diffusion. This results from the combined effects of a defect large relative to the tip dimensions and the limited transport process due to diffusion. The instabilities found in the graph also reflect the rough topography of the sample surface compared to the tip dimensions. Finally, the stable electrochemical performance of the tip throughout the measurements is confirmed by the cyclic voltammograms shown in Figure 10B, that were recorded after measuring each scan line in Figure 10A.

**Figure 11:**
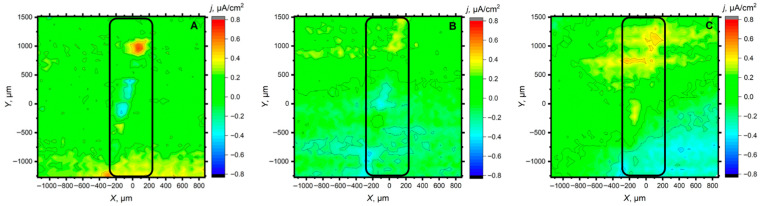
SVET maps of a mild steel substrate coated with a commercial epoxy coating containing alumina particles loaded with 8HQ at various times of exposure: (**A**) 5 min, (**B**) 120 min and (**C**) 300 min after immersion in 0.5 M NaCl solution. Tip-substrate distance: 100 μm; tip vibration frequency: 70 Hz in the vertical axis; amplitude of tip vibration: 20 μm.

**Figure 12:**
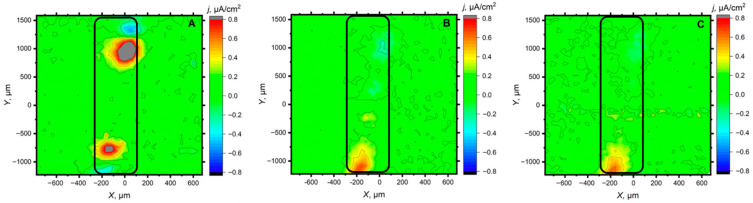
SVET maps of a mild steel substrate coated with a commercial epoxy coating containing alumina particles loaded with BTA at various times of exposure: (**A**) 5 min, (**B**) 120 min and (**C**) 300 min after immersion in 0.5 M NaCl solution. Tip-substrate distance: 100 μm; tip vibration frequency: 70 Hz in the vertical axis; amplitude of tip vibration: 20 μm.

Since the scan lines did not allow to verify the representativeness of the behavior over the entire defect, these last two systems were then analyzed using the scanning vibrating electrode electrochemical technique (SVET). Although its spatial resolution is lower, it allows to monitor a large portion of the scratched area in a single scan. A selection of the obtained SVET maps is shown in Figure 11 and Figure 12 for the same time interval as in the SECM studies of Figure 7. It is observed that in both cases, the metal in the scratch is initially activated, with the formation of one (or two) very localized anodic region corresponding to the formation of corrosion pits, with most of the exposed metal in the defect acting as a cathode for oxygen reduction. However, over time, the magnitude of both the anodic and cathodic processes decreases significantly, indicating that the loaded inhibitors have been released from the alumina particles into the defect, which consequently slows down the progression of the corrosive process on the exposed steel. The protective effect appears to be more effective in the case of 8HQ than for BTA at 300 min of exposure.

**Figure 13:**
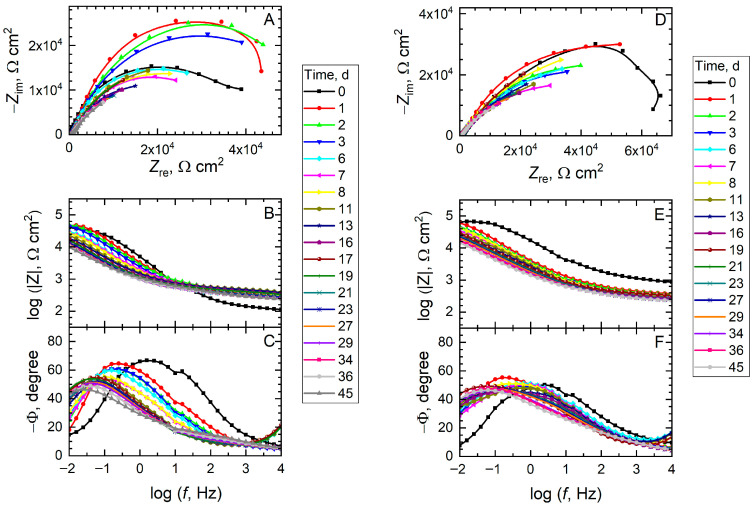
Electrochemical impedance spectra (EIS) of mild steel coated with a commercial epoxy coating during exposure to 0.5 M NaCl solution. A scratch was produced to the coating before immersion in the test environment. Type of coating: (**A**–**C**) unmodified; (**D**–**F**) containing alumina particles without functionalization with a corrosion inhibitor.

**Figure 14:**
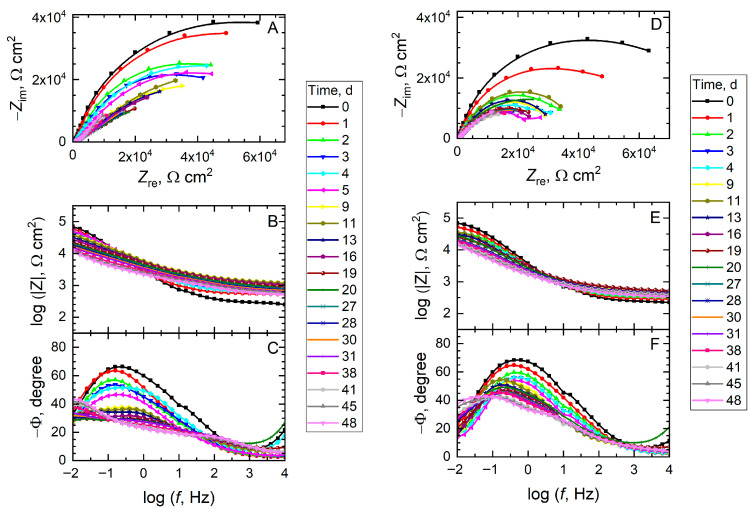
Electrochemical impedance spectra (EIS) of mild steel coated with a commercial epoxy coating during exposure to 0.5 M NaCl solution. A scratch was produced to the coating before immersion in the test environment. Type of coating: (**A**–**C**) functionalized with alumina particles filled with 8HQ; (**D**–**F**) functionalized with alumina particles filled with BTA.

The differences in corrosion protection of damaged mild steel coated with the four coating systems were also investigated using electrochemical impedance spectroscopy (EIS). Although the electrochemical response obtained with this technique lacks spatial resolution because it averages the current flowing over the entire exposed surface, it allows for the study of effects on a larger scale, more comparable to the expected behavior of the systems under real operating conditions. The impedance spectra measured at different immersion times in 0.5 M NaCl solution are shown in Figure 13 and Figure 14 as Nyquist and Bode plots. A cursory inspection of the spectra allows the following observations to be deduced. First, none of the spectra exhibits a capacitive range corresponding to a barrier coating, with phase angles close to −90°, indicating that the electrochemical response is dominated by the corrosion process occurring in the scratch following direct exposure of the mild steel surface to the chloride-containing aqueous solution. Second, the magnitude of the impedance and a more resistive behavior are observed over time, even resulting in spectra that do not exhibit a capacitive-resistive transition (which occurs at a phase angle of −45°), these effects being particularly visible for the spectra in Figure 13 corresponding to coatings not functionalized with corrosion inhibitors. In contrast, active dissolution of the metal proceeds at a slower rate in the case of the spectra in Figure 14, with BTA providing a more lasting corrosion protection effect. And third, although some dispersion in the low-frequency range can be observed upon examination of the Nyquist plots, the electrochemical activity in the impedance spectra mostly results in the development of a single semicircle, indicating that only one time constant is active at any instant.

**Figure 15:**
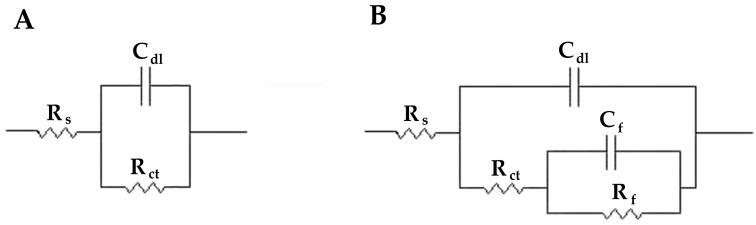
Equivalent circuits (EC) employed to fit the electrochemical impedance spectra of Figure 13 and Figure 14. (**A**) Simplified Randles’ circuit describing a freely corroding metal surface, and (**B**) equivalent circuit describing a metal surface covered by a non-sealing surface layer.

This behavior is consistent with the initiation of a metal dissolution reaction in the bare metal, which can be described by a polarization resistance and a double-layer capacitance using the simplified Randles’ equivalent circuit (EC) shown in Figure 15A [49]. This EC includes the uncompensated resistance of the test electrolyte and electrical connectors (R_S_), the polarization resistance of the substrate (R_ct_), and the double-layer capacitance at the substrate/electrolyte interface (C_dl_).

In the case of the coatings containing MA particles, loaded with inhibitors, and for immersion periods longer than one month, the low-frequency dispersion observed in the Nyquist diagrams of Figure 14 can be better adjusted by introducing a second time constant in the equivalent circuit, as shown in Figure 15B. This circuit has been used to describe the electrochemical behavior of a non-sealing film or layer deposited on the surface of a corroding metal [50]. Then, this second time constant results from the development of two different current paths in the system: one corresponding to the bare metal-electrolyte interface, and the other corresponding to the presence of a surface film or a layer of corrosion products partially covering the metal within the scratch. In this circuit, R_f_ describes the resistance and C_f_ the associated capacitance of the surface layer, respectively.

Additionally, to better fit the spectra, constant phase elements (CPE) were used instead of pure capacitances because depressed semicircles are observed in the Nyquist diagrams of Figure 13 and Figure 14. The impedance representation of a CPE is given in terms of a constant *Y*_0_ and an exponent *n* indicating the deviation from ideal capacitive behavior, and the capacitance values are determined from these CPE parameters using the following equation [51]:

(4) $$C={\left(R^{1-n}Y_{0}\right)}^{1/n}$$

Table 3 and Table 4 give the impedance parameters determined using these equivalent circuits to model the experimental impedance spectra of Figure 13 and Figure 14.

**Figure 16:**
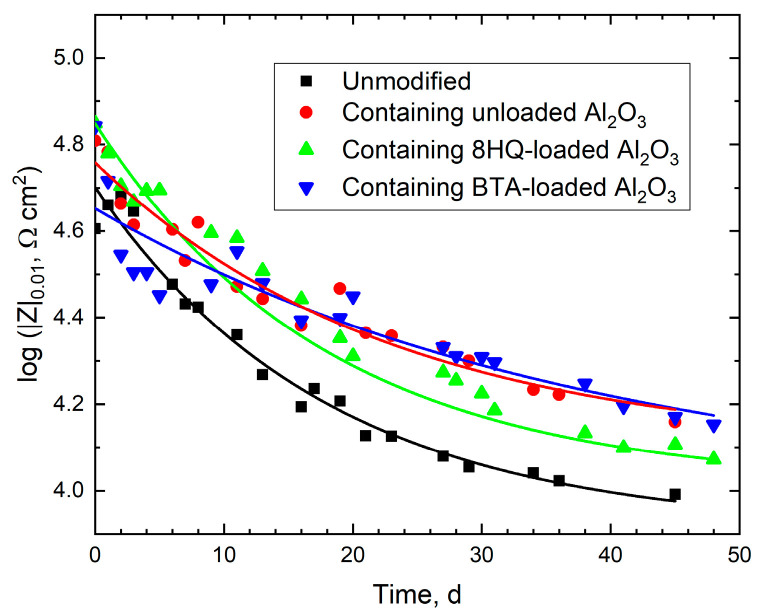
Time evolution of the impedance amplitude at the low-frequency (0.01 Hz) limit of the impedance spectra in Figure 13 and Figure 14. Coating systems: commercial epoxy matrix without additives (black), epoxy matrix with: unloaded (red), 8HQ-loaded (green) and BTA-loaded (blue) MA particles. Lines represent a decaying exponential fit through the experimental data for easier comparison.

For easier comparison, Figure 16 shows the time evolution of the impedance amplitude at the low-frequency limit of the spectra in Figure 13 and Figure 14 for the four coating systems. A significantly improved corrosion resistance of mild steel exposed to artificial damage is observed when the coating contains alumina particles, due to their leaching through the damaged walls at the scratch. More importantly, functionalizing the coating with a corrosion inhibitor effectively contributes to slowing down the corrosion rate of the metal, although with different behaviors for 8HQ and BTA. Therefore, the restorative action of 8HQ occurs more rapidly than that of BTA, especially during the first week of exposure, which is consistent with the observations obtained using microelectrochemical techniques with very short exposures in Figure 7, Figure 10 and Figure 11. Unfortunately, the beneficial effect of 8HQ diminishes over time, while that of BTA is more stable, the latter being a better choice for longer exposures. These observations can be summarized by defining an inhibitory efficiency value for a given exposure time. In this case, the values of |Z|_0.01_ measured after 42 days for each system were selected and compared to the value presented by the unmodified coating system using the expression:

(5) $$\%=\frac{Z_{0}{-Z}_{i}}{Z_{0}}\times 100$$

where the indices “0” and “*i*” denote the unmodified and inhibited systems, respectively [52]. The resulting inhibitory efficiency values are listed in Table 5.

**Figure 17:**
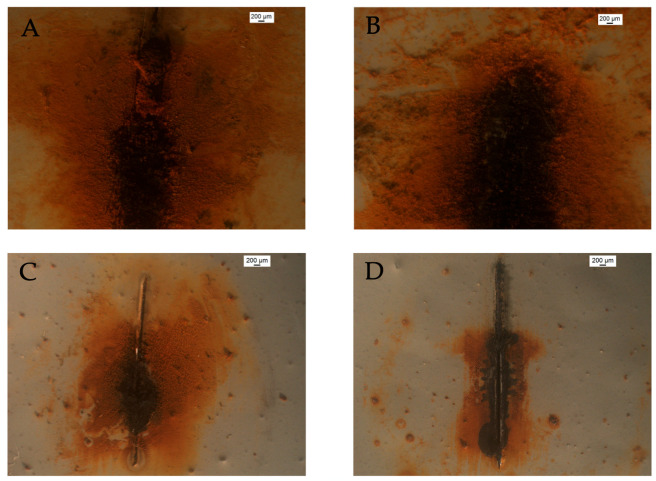
Optical microscope images showing the scratched surface of the painted mild steel after completing the EIS measurements shown in Figure 13 and Figure 14. Coating systems: (**A**) unmodified coating, (**B**) coating containing alumina particles, (**C**) coating containing particles loaded with 8HQ, and (**D**) coating containing particles loaded with BTA.

In addition, by examining the surface state of the damaged coated samples after the impedance tests, observed under an optical microscope, complementary information to the electrochemical results can be obtained. Figure 17 shows the corresponding micrographs.

The comparison between the electrochemical results in Figure 16 and the surface observations in Figure 17 establishes a clear correlation between coating performance and the final condition of the damaged areas. In the unmodified system (Figure 17A), the pronounced decrease in impedance over time (see black line in Figure 16) translates into extensive formation of corrosion products around the scratch, evidencing the absence of significant protective action. In contrast, the inhibitor-functionalized coatings (Figure 17C,D) show far less severe deterioration, consistent with the enhanced corrosion resistance reflected in the impedance values. In particular, coating containing particles loaded with BTA maintains the highest level of protection over prolonged exposure times, as confirmed by the elevated impedance in Figure 16 and the limited oxide growth within the scratch in Figure 17D. This validates the sustained release of the inhibitor and its effectiveness under long-term exposure. Unlike 8HQ-loaded MA, which shows a more immediate protective effect, BTA tends to be released more slowly from the alumina particles, with its advantage becoming more apparent at longer times, when sustained release prevents further impedance decay.

Although the coating containing unloaded MA particles exhibit an electrochemical behavior close to that shown by the coating with the BTA-loaded ones in terms of the impedance values in Figure 16, the images in Figure 17B,D reveal a clear difference in the state of the scratched samples at the end of the experiment. The former displays abundant accumulation of corrosion products around the defect, whereas the latter shows a visually cleaner scratch. This discrepancy can be explained by a combination of effects. On the one hand, in the absence of inhibitor, the anodic dissolution of steel proceeds actively, releasing Fe^2+^ ions that subsequently precipitate as hydroxides/oxides, which accumulate and become visible on the surface (Figure 17B). On the other hand, when MA are loaded with BTA, the local release of the inhibitor promotes the formation of a stable adsorbed film on steel, suppressing anodic dissolution and thereby preventing oxide accumulation within the scratch. Additionally, the surface chemistry of alumina (≡Al–OH groups) and micrometric pH variations may modulate both the efficiency and precipitation pathway of corrosion products. That is, since the hydrolysis of soluble aluminum species to form [M(OH)]^(n−1)+^ complexes is less acidic than that of iron [53], the acidity of the electrolytic environment within the scratch will be less in the case of the coating modified with the MA particles, thus leading to smaller metal dissolution rates. In addition, the higher pH values also facilitate the precipitation of corrosion products within the scratch to a greater extent. As result, the greater surface deterioration observed for unloaded alumina most likely reflects not only the absence of chemical protection but also a reservoir effect that enhances electrolyte retention, favoring corrosion processes, while simultaneously providing a partial physical barrier against electrolyte penetration and further metal dissolution. Indeed, it has been shown elsewhere that the addition of alumina particles reduces the corrosion current density (*i*_corr_) and increases corrosion resistance [12,54], likely due to a reduction in infiltration pathways for aggressive species within the coating film.

In summary, the alumina particles synthesized using an electrochemical route demonstrated adequate capacity to contain corrosion inhibitors as to functionalize organic coatings for the corrosion protection of metal substrates.

## 4. Conclusions

Electrochemical technology for the synthesis of mesoporous alumina particles has been developed, which is easy to implement. In fact, the electrochemical system is used for the production of the chemical reagents themselves as well as their removal by simple separation procedures such as filtration and decantation. The electrochemical interface is also quite simple, consisting solely of a current source with the only requirement of potential reversal capabilities, so that it is possible for the aluminum electrodes to regularly exchange their functions as cathodes and anodes in the electrochemical cell, thus effectively avoiding system failure by electrode passivation.

In addition to its technical advantages, electrocoagulation offers practical benefits in terms of cost and environmental considerations. Unlike sol–gel or hydrothermal methods, which demand specialized equipment, elevated temperatures, or the use of organic solvents, electrocoagulation operates under mild conditions, typically at room temperature and in aqueous media, thereby minimizing energy consumption and avoiding hazardous chemicals (with tap water and aluminum electrodes as the only required components). The process is straightforward to scale up, requires relatively low capital investment, and can be powered by renewable energy sources [55]. These characteristics emphasize electrocoagulation as a cost-effective and environmentally sustainable synthesis route, making it particularly attractive for practical applications [54].

Furthermore, the conversion of the wet sludge obtained from the electrochemical cell into suitable MAs is achieved by a sequence of heating stages that involve drying and calcining that can be easily performed in a conventional electric oven. Any typical procedure can then be used to load the particles with a corrosion inhibitor, resulting in loading ratios comparable to those obtained using MA particles produced by more complex synthesis routes.

Essentially, this new synthesis route also overcomes several drawbacks associated with the use of other metal oxide particles. For instance, although mesoporous silica has been widely investigated, it requires additional functionalization steps, such as silylation, to stabilize the corrosion inhibitors within the porous framework; these steps increase cost and complexity [15]. Similarly, TiO_2_-based containers, despite their popularity, have lower specific surface areas and present photocatalytic activity, which can compromise the stability of the coating under UV light. In contrast, MA offers superior chemical stability, higher hardness, and in some cases, a specific surface area up to 20 times greater than that of TiO_2_ [16]. Therefore, alumina acts not only as a passive filler that blocks micropore pahtways and delays electrolyte ingress through an intact coating [10,56], but also works as an active container [12,16].

To validate this approach, a proof of concept of its applicability to obtain active corrosion protection coating systems was demonstrated by functionalizing a commercial epoxy formulation recommended for marine applications. The alumina particles were successfully loaded with 8HQ or BTA as corrosion inhibitors and subsequently dispersed in the epoxy matrix to produce the functionalization of the anticorrosion organic coating.

Corrosion analysis by EIS allowed monitoring changes in electrochemical reactivity of coated carbon steel around an artificial scratch that were related to the addition of loaded and unloaded MA to the polymer matrix. Although the best corrosion performance was initially obtained in the case of alumina particles filled with 8HQ, BTA-loaded Al_2_O_3_ provided a more durable protection of mild steel in a simulated marine environment.

Overall, the results of this study provide relevant information for the development of next-generation protective coatings in industrial sectors where metallic infrastructures are continuously exposed to aggressive saline environments. The electrochemical synthesis route described here is simple, inexpensive, and environmentally friendly, making it a promising alternative for the large-scale production of MA containers. Moreover, the demonstrated ability of the particles to act simultaneously as mechanical reinforcements and as containers for the controlled release of corrosion inhibitors offers dual functionality that could reduce the need for multiple additives in commercial epoxy formulations. This multifunctional performance is particularly interesting for extending the service lifetime, reducing maintenance costs, and mitigating the environmental impact of anticorrosion strategies, thus highlighting the potential industrial relevance of integrating MA-based additives into existing coating technologies.

## Figures and Tables

**Table 1:** BET parameters of the MA particles.

BET Data		
Surface area	BET area	231 m^2^/g
	Langmuir surface area	350 m^2^/g
Pore volume	Pore volume	443 mm^3^/g
	Pore size	7.68 nm

**Table 2:** Dimensions of the artificial scratches produced on coated steel samples for corrosion studies, including average values and standard deviations. The coating composition was modified by the addition of MA particles with and without the addition of corrosion inhibitors. Measurements were performed with an optical microscope and used to normalize subsequent electrochemical data.

Sample	Length, mm	Width, mm	Area, mm^2^	Average Length, mm	Average Area, mm^2^	Standard Deviation
Unmodified coating	5.63	0.05	0.28	5.51	0.28	0.02
Unloaded Al_2_O_3_	5.14	0.05	0.26			
8HQ-loaded Al_2_O_3_	5.29	0.05	0.26			
BTA-loaded Al_2_O_3_	5.98	0.05	0.30			

**Table 3:** Impedance parameters fitted to the impedance spectra of Figure 13 corresponding to mild steel coated with a commercial epoxy coating during exposure to 0.5 M NaCl solution. The coating was either applied as such or modified with non-functionalized MA particles. A scratch was produced in the coating before immersion in the test environment.

Unmodified Coating			Coating Containing Alumina Particles		
t, days	$R_{\mathrm{ct}},\;k\Omega$ cm^2^	C_dl_, µF/cm^2^	t, days	$R_{\mathrm{ct}},\;k\Omega$ cm^2^	C_dl_, µF/cm^2^
1	48.4 ± 6.8	202 ± 11	1	91.8 ± 7.9	407 ± 13
2	58.6 ± 10.1	330 ± 14	2	64.1 ± 4.8	509 ± 14
3	53.3 ± 9.5	395 ± 16	3	57.2 ± 3.4	572 ± 14
6	31.5 ± 2.6	509 ± 14	6	62.5 ± 4.4	636 ± 17
7	30.6 ± 2.5	693 ± 25	7	44.1 ± 2.1	535 ± 11
8	34.7 ± 3.9	908 ± 38	8	75.2 ± 5.5	740 ± 16
11	42.6 ± 6.8	1666 ± 69	11	52.5 ± 3.0	1003 ± 20
13	41.9 ± 8.2	2522 ± 104	13	62.8 ± 4.4	1684 ± 30
16	39.5 ± 10.8	3888 ± 155	16	47.7 ± 2.8	1651 ± 20
19	42.4 ± 5.9	3055 ± 88	19	89.2 ± 10.3	2356 ± 50
21	27.8 ± 2.7	1275 ± 190	21	49.5 ± 3.3	2073 ± 36
23	26.9 ± 3.8	1388 ± 271	23	62.3 ± 10.2	2808 ± 57
27	24.3 ± 2.6	1589 ± 272	27	55.2 ± 6.0	2840 ± 56
29	25.8 ± 4.0	1850 ± 355	29	43.8 ± 4.3	2561 ± 54
34	29.5 ± 5.6	1906 ± 379	34	44.4 ± 5.0	4044 ± 92
36	24.7 ± 2.9	1957 ± 315	36	50.9 ± 7.9	5523 ± 140
42	34.1 ± 8.5	2373 ± 481	42	58.1 ± 15.4	10,104 ± 282

**Table 4:** Impedance parameters corresponding to the impedance spectra in Figure 14, corresponding to mild steel coated with a commercial epoxy coating containing MA particles loaded with corrosion inhibitors. The measurements were carried out when the sample was exposed to a 0.5 M NaCl solution. A scratch was produced in the coating before immersion in the test environment.

Coating Containing Alumina Particles Filled with 8HQ					Coating Containing Alumina Particles Filled with BTA				
t, days	$R_{\mathrm{ct}},\;k\Omega$ cm^2^	C_dl_, µF/cm^2^	$R_{c},\;k\Omega$ cm^2^	C_c_, µF/cm^2^	t, days	$R_{\mathrm{ct}},\;k\Omega$ cm^2^	C_dl_, µF/cm^2^	$R_{c},\;k\Omega$ cm^2^	C_c_, µF/cm^2^
1	63.7 ± 4.1	312 ± 14			1	49.6 ± 2.5	198 ± 5		
2	57.5 ± 4.7	374 ± 12			2	32.4 ± 1.6	220 ± 8		
3	53.1 ± 3.3	384 ± 11			3	27.1 ± 1.6	240 ± 9		
5	58.0 ± 2.9	350 ± 7			5	20.3 ± 1.8	238 ± 11		
9	69.2 ± 4.9	1691 ± 36			9	25.9 ± 1.6	291 ± 12		
13	67.3 ± 6.0	2109 ± 45			13	26.9 ± 2.1	322 ± 14		
16	93.3 ± 7.9	7216 ± 84			16	230 ± 1.3	399 ± 15		
19	84.1 ± 6.9	12,860 ± 130			19	25.9 ± 1.7	508 ± 18		
38	6.92 ± 3.07	559 ± 76	87.5 ± 54.1	676 ± 150	38	6.89 ± 3.01	555 ± 72	89.1 ± 54.1	681 ± 147
41	2.21 ± 0.80	67.5 ± 18.8	37.0 ± 8.9	1338 ± 236	41	2.23 ± 0.79	68.7 ± 18.3	37.7 ± 9.2	1316 ± 227
45	2.65 ± 0.87	86.6 ± 20.1	60.1 ± 21.8	1606 ± 252	45	2.67 ± 0.84	87.7 ± 19.3	61.2 ± 21.9	1593 ± 241
48	2.11 ± 0.60	68.8 ± 16.6	51.7 ± 16.6	1811 ± 242	48	2.13 ± 0.57	70.9 ± 16.0	52.6 ± 16.3	1788 ± 228

**Table 5:** Inhibitory efficiency values determined from the low-frequency impedance values measured after 42 days exposure in Figure 16.

Sample	% Inhibitory Efficiency
Unmodified coating	-
Unloaded Al_2_O_3_	46.1
8HQ-loaded Al_2_O_3_	30.4
BTA-loaded Al_2_O_3_	50.1

## Data Availability

The original contributions presented in this study are included in the article. Further inquiries can be directed to the corresponding authors.
